# Commentary: Why Cognitive Behavioral Therapy Is the Current Gold Standard of Psychotherapy

**DOI:** 10.3389/fpsyt.2019.00123

**Published:** 2019-03-12

**Authors:** Héctor Fernández-Álvarez, Javier Fernández-Álvarez

**Affiliations:** ^1^AIGLÉ Foundation, Buenos Aires, Argentina; ^2^Department of Psychology, Catholic University of Sacred Heart, Milan, Italy

**Keywords:** integration, integrative cognitive behavioral therapy, CBT (cognitive-behavioral therapy), information processing, evidence based process

We read with great interest the commentary by David et al. (1) in which the authors give a series of reasons to support that cognitive behavioral therapy (CBT) constitutes the current *gold standard* of psychotherapy. Their main argument revolves around the fact that CBT provides the most solid evidence in terms of efficiency (both efficacy and effectiveness). The authors back up this idea, principally, by providing an accurate description of the methodological strengths of CBT and finally by presenting some conceptual arguments. Although we broadly agree with the three described reasons outlined by the authors, we consider it important to incorporate some additional ideas to this discussion.

Our principal interest is to extend the discussion regarding the integrative efforts that are being carried out in our field. We are firmly convinced that CBT constitutes the most appropriate approach to foster the integration among the different psychotherapeutic theoretical approaches (2). Psychotherapy is still diving into pre-paradigmatic waters, and the integration of theories is a rather logical goal that any scientific discipline that aspires to reach a paradigmatic stage should set (3). As some authors explained elsewhere (4, 5), the mere accumulation of empirical evidence is not enough to demonstrate the theoretical consistency of an approach. In other words, the empirical evidence of the benefits of the CBT does not ensure its theoretical foundations (6). By theoretical consistency, we understand not only “*evidence based on (1) experimental studies (and sometimes additional/adjunctive correlational studies) and/or (2) component analyses, patient–treatment interactions, and/or mediation/moderation analyses in complex clinical trials (CCTs)”* (4), but also epistemological consistency to add to an accurate conceptual debate.

Cognitive therapy (CT) history can be characterized as a continuity of tensions derived in an ever-growing integration of its theoretical corpus and practical tools. Overall, we identify three tensions, described in detail elsewhere (1), which resulted in the development of integrative efforts, which greatly enriched the field of psychotherapy. First, pure CT extended not only to many clinical conditions, but also to other integrative approaches, like Dialectic Behavior Therapy [DBT; (7)] or the Cognitive Analytic Therapy [CAT; (8)]. Later, Alford and Beck (9) explicitly proposed that CT, as an approach that is technically eclectic and theoretically solid due to the consistency of the cognitive theory, could be adopted as a solid scientific foundation fostering a common language for the clinical practice. As a consequence, certain behavioral techniques, primarily exposure, became central procedures of CT. Subsequently, rapidly cognitive and behavioral therapies were integrated into the current CBT.

Finally, further integrative development can be identified when fierce critics claim that CBT was based on a realistic epistemological framework using interventions centered mainly on contents instead of functions and directing the therapeutic goals to a mere reduction of symptoms. Although this tension initially provoked a considerable dispute (10), acceptance, and commitment therapy and all the associated techniques that gained strength with this movement (e.g., mindfulness, decentering, flexibility processes, etc.) are currently smoothly incorporated into the CBT corpus (11).

The integration of a field does not imply the elimination of the components or the parts but their articulation. The proposal of adopting a program to integrate the different models and approaches that form the field of psychotherapy does not respond only to the natural tendency to integration that is observed in any field of science, but in the field of psychotherapy also as the most favorable option to address clinical phenomena by virtue of its extraordinary complexity. At the same time, the search for integration must be understood as an open research program. In this sense, it is expected that the evolution of psychotherapy and the emergent progressive theoretical and technical developments require proposals for progressive integration that would account for this growing complexity. A program to integrate psychotherapy should not be understood as a task with conclusive ends but as a persistent way of responding to the new questions raised by the discipline.

We understand this ongoing integrative effort is 2-fold:

1. It embraces the possibility of expanding the cognitive theory to support the field in line with the aforementioned ideas of Alford and Beck. In that sense, some of the most important epistemological objections to CBT focus on the representational nature of thoughts and beliefs. Representational theories of cognition consider beliefs and thoughts to be causally efficacious mental representations of facts, states of affairs, or propositions. Critics of representationalism have argued that CBT confuses individuals' thought reports (which are represented as having imagistic or linguistic content) with their thoughts (which need not have any distinct representational content at all) (12).
  In order to progress in this regard, it is important to emphasize that the theoretical structure of CBT is permeable to employ a model of the theory of mind capable of taking into account not only the information processing paradigm in formal and logical terms, but also the diverse modalities of this paradigm. Nowadays, we count on a myriad of models developed within the cognitive sciences to explain the different modalities in which the reality is processed more accurately. Among those models, embodied cognition, situated cognition, extended cognition, or dynamic cognition should be mentioned (13). Each of these modalities can be articulated with the different theoretical approaches that constitute the other principal axes of psychotherapy: the psychodynamic approach, the humanistic approach, and the systemic approach.
2. The general consensus on the fact that CBT, along with the systemic, the humanistic-existential, and the psychodynamic approaches conform to the four main traditions in which the whole theoretical corpus of psychotherapy can be organized has been increasing (14). In the same way as CT and behavior therapy were successfully integrated into an integrated approach with multiple manifestations, we believe that it is possible, expectable, and potentially beneficial to consider the broad cognitive-behavioral avenue as an assimilative channel that may operate as an articulating axis of the different approaches. Hence, it is necessary to develop a model that does not attempt to act in a reductionist manner with respect to the other approaches but to lean on them and move toward a theoretical elaboration that has certain fundamental organizing principles as its axis. In that sense, following the characterization of scientific progress offered by Kuhn (15) and used by David et al.(4), we believe that in order to reach a state of normal science, the integration of the four main theoretical models should be attempted.

Indeed, many examples throughout history of psychotherapy have tried to illustrate how integration can be achieved in any of the four main traditions of integration: theoretical integration, assimilative integration, eclectic integration, and common factors (16). Likewise, apart from the main integrative tradition, multiple integrative expressions are not necessarily recognized in any of those four roots; nevertheless, a genuine and successful integration was achieved. By way of illustration, Hayes (17) maintained that acceptance and commitment therapy has a strong connection with the general principles of humanistic-existential therapy. In the same vein, motivation interviewing or systemic approaches have been widely adopted within CBT treatments (18). As another illustrative example, DBT and mentalization therapy are increasingly considered to be equally effective (19) and even similarly conceptualized (20).

## Why CBT as the Central Organizing Axis of Integration?

CBT is conceptually organized around a strongly connectionist model of explanation that permits to articulate the different approaches on a common axis in which the diverse mental operations can be integrated into a hierarchical schema. In turn, this schema can account for the multiple levels of organization that characterizes the architecture of mental phenomena. Such a model would enable the integration of the two ways in which the processes within psychotherapy are deployed and organized in order to find adequate therapeutic designs and more effective interventions. These two dimensions are the behavior and the experience, which are developed simultaneously at different levels. Both should be considered, given that patients' demands and needs always require addressing both facets.

In other words, three fundamental aspects can foster the integration of the field: (a) a broad information processing framework, (b) interventions focused both on the behavioral and experiential level, and (c) a psychopathological model organized around the central role of personality.

Apart from our own model that seeks to provide this kind of integrated psychopathological understanding and therapeutic intervention (2, 21), many other expressions reflect this philosophical and practical standpoint in different ways.

Going beyond the classical cognitive model, we can mention the research program brought forth by Sander Koole and colleagues. Their cognitive perspective is integrated with principles of embodied and situated cognition, which permits to grasp the clinical phenomenon (i.e., emotion regulation) from a wider perspective (22).

Another illustrative example that directly focused on psychotherapy procedures is constituted by the Methods of Levels [MOL; (23)]. MOL is based on the Perceptual Control Theory, and it focuses on the two aforementioned levels: (control of) behaviors and experience.

In turn, a psychopathological model that places personality in a central role to enable a solid diagnosis and prognosis of the clinical situation permits us to reach an adequate articulation between behaviors and ways of organizing the experience (functional or dysfunctional). An illustrative example of how this integration can be achieved is the cybernetic framework of personality proposed by De Young, which takes principally into account the study of goal-directed self-regulating systems (24).

We acknowledge the difficulty in establishing a model capable of addressing simultaneously nomothetic and ideographic aspects that define the mental functioning and clinical situation (context/practice). Thus, it is greatly intricate to translate this complexity into specific empirical research lines. However, our proposal is that CBT should integrate not only the empirical evidence, but also the epistemological solidity that facilitates articulation of the different levels that conform to the ontological status of the mind in the best possible way.

In summary, we celebrate the integrative spirit that is starting to arise within the CBT community. Nonetheless, we believe it is essential to conceptualize psychotherapy still as a pre-paradigmatic discipline that could greatly benefit from a deep discussion to solidly integrate the main therapeutic approaches. In that sense, we strongly advocate for adding the epistemological discussion to the empirical one in order to support CBT as the pivot in this path toward integration.

## Author Contributions

HFÁ and JFÁ equally contributed to the conception of the article, working together in the draft until the completion of the work. Both authors revised and approved the submitted version of the manuscript.

### Conflict of Interest Statement

HFÁ is the honorary president of AIGLÉ Foundation, a NGO clinical center committed to the delivery and dissemination of integrative psychotherapy. The remaining author declares that the research was conducted in the absence of any commercial or financial relationships that could be construed as a potential conflict of interest.
